# Potentials and barriers in cooperation between conventional and complementary practitioners at a Danish Multiple Sclerosis Hospital

**Published:** 2011-09-27

**Authors:** Lasse Skovgaard

**Affiliations:** The Danish Multiple Sclerosis Society, Mosedalvej 15, 2500 Valby, Denmark; Department of Public Health, University of Copenhagen, Øster Farimagsgade 5, 1014 Copenhagen K, Denmark; National Research Center in Complementary and Alternative Medicine, NAFKAM, Faculty of Health Science, University of Tromsø, N – 9037 Tromsø, Norway

**Keywords:** multiple sclerosis, integrative treatment, integrative care, teambased treatment, complementary medicine, CAM

## Abstract

**Introduction:**

More than 50% of the People with Multiple Sclerosis (PwMS) in Denmark have used complementary and alternative medicine (CAM). The majority of these combine CAM and conventional treatment, and from 2004 to 2010 the Danish Multiple Sclerosis Society conducted a research project with the purpose of investigating and testing models for cooperation between conventional and complementary practitioners.

**Theory:**

Five health care providers and five CAM practitioners were set up to work together from 2004 to 2010 in developing and offering integrated treatment to 200 PwMS at a Danish MS Hospital. The investigation of the collaborative process between practitioners has been based on theories of epistemic cultures as well as learning theories, focusing on interdisciplinary development.

**Methods:**

Empirical material consists of individual interviews with practitioners, a group interview with practitioners, a group interview with professional staff at the Danish MS Hospital, interviews with patients as well as written responses from the practitioners.

**Results and conclusions:**

The six-year cooperation among the practitioners documented mutual inspiration and learning within the team, as well as numerous basic challenges involved in developing interdisciplinary treatment for PwMS. Cooperation between researchers and the treatment team resulted in the development of four interdisciplinary models, which describe the potentials and barriers in relation to various types of collaboration.

**Discussions:**

In many cases, integrating CAM and conventional treatment providers is seen as an ideal. This paper points out the importance of not overlooking the opportunities, values and the potential inherent in a pluralistic ideal in terms of the patients’ own active efforts and the dynamism that can arise when the patient becomes a co-informant, co-coordinator and/or co-integrator.

11^th^ Annual Integrated Care Conference, Odense, Denmark, March 30–April 1 2011

